# Airborne Quantum Key Distribution Performance Analysis under Supersonic Boundary Layer

**DOI:** 10.3390/e25030472

**Published:** 2023-03-08

**Authors:** Huicun Yu, Bangying Tang, Haolin Ding, Yang Xue, Jie Tang, Xingyu Wang, Bo Liu, Lei Shi

**Affiliations:** 1 Information and Navigation College, Air Force Engineering University, Xi’an 710077, China; 2 College of Advanced Interdisciplinary Studies, National University of Defense Technology, Changsha 410073, China; 3 College of Computer and Science, National University of Defense Technology, Changsha 410073, China; 4 College of Aerospace Science and Engineering, National University of Defense Technology, Changsha 410073, China; 5 Academy of Military Sciences, Beijing 100864, China

**Keywords:** boundary layer, quantum key distribution, airborne

## Abstract

Airborne quantum key distribution (QKD) that can synergize with terrestrial networks and quantum satellite nodes is expected to provide flexible and relay links for the large-scale integrated communication network. However, the photon transmission rate would be randomly reduced, owing to the random distributed boundary layer that surrounding to the surface of the aircraft when the flight speed larger than Mach 0.3. Here, we investigate the airborne QKD performance with the BL effects. Furthermore, we take experimental data of supersonic BL into the model and compare the airborne QKD performance under different conditions. Simulation results show that, owing to the complex small-scale turbulence structures in the supersonic boundary layer, the deflection angle and correspondingly drifted offset of the beam varied obviously and randomly, and the distribution probability of photons are redistributed. And the subsonic and supersonic boundary layer would decrease ~35.8% and ~62.5% of the secure key rate respectively. Our work provides a theoretical guidance towards a possible realization of high-speed airborne QKD.

## 1. Introduction

Quantum key distribution (QKD) is a promising technology to realize secure transmission, the fundamental principles of quantum mechanics guarantee the safety of QKD, which ensures the generation of information-theoretical-secure keys for distant users [1,2,3,4,5]. Furthermore, QKD can resist eavesdropping detection and tamper relying on physical principles, which has tremendous utilization potential in terms of finance, government, and military [6,7]. Up to now, remarkable progress has been achieved both in fiber links [8,9,10,11] and quantum satellites [12,13,14,15,16,17,18,19] QKD systems, and gradually transferred from the laboratory to realistic applications. However, with constant orbits, limited communication time window and night-only quantum satellites, airborne [20,21,22,23,24,25,26,27] platforms are ideal mobile nodes that can synergize with terrestrial links and quantum satellites to build the mobile, on-demand, and real-time coverage quantum network. For example, airborne quantum nodes could serve as temporary relays to solve the last-mile quantum key exchange for an inner-city or a field network benefiting from their rapid deployment capabilities.

Several groups have reported their research in airborne quantum communications in recent years, the first demonstration was implemented in 2013, with the platform flying at the speed of 290 km/h and a height of 1.1 km [20].

In 2020, Nanjing University accomplished the first drone-based entanglement distribution [23] and achieved 200 m coverage and duration of 40 min, and in the next year, they expand the distance to 1000 m by means of one more drone [21] as an optical-relay. Compared to satellite quantum communication, airborne QKD features in high-speed maneuverability and suffers from the complicate atmosphere environment, including atmospheric turbulence, background noise and attitude disturbance, worse still, the boundary layer (BL) would also severely impact the quantum signal transmission. When the aircraft is flying at a high speed, usually larger than 0.3 Ma, a very thin layer of air will stick over the surface of the aircraft with high velocity, resulting in the BL, and it would introduce random disturbance to the transmitted photons, which would reduce the coupling efficiency and fidelity of quantum states [28], and decrease the performance of aircraft-based QKD. Currently, most implementations of airborne QKD only analyzed the noise from atmospheric turbulence and molecular scattering [25,26], but ignored the boundary layer effects. In 2021, our group proposed an airborne QKD performance evaluation scheme that takes the boundary layer effect into account. The analysis results show that the aerodynamic optical effects caused by the boundary layer should be attended to, which will greatly reduce the final key rate [29].

In this article, an airborne QKD performance evaluation scheme with supersonic BL effect is proposed. We first simulate the density and refractive index field of the BL. Then, the photon propagation model in the boundary layer is established by performing the ray tracing method that uses the Adams linear multistep method. In the end, we present the analysis results about the key rate of photons transmitted through the measured supersonic BL. Owing to the complex small-scale turbulence structures in the supersonic boundary layer, the deflection angle and correspondingly drifted offset of the beam varied obviously and randomly, and the distribution probability of photons is redistributed. The result shows that the subsonic and supersonic boundary layer would decrease ~35.8% and ~62.5% of the secure key rate respectively. With the increase of speed *v*, the key-rate curve is obviously jittering, and the QKD performance is continuously reduced. Our work provides a theoretical guidance towards a possible realization of high-speed airborne QKD.

## 2. Method

### 2.1. Background

**Figure 1:**
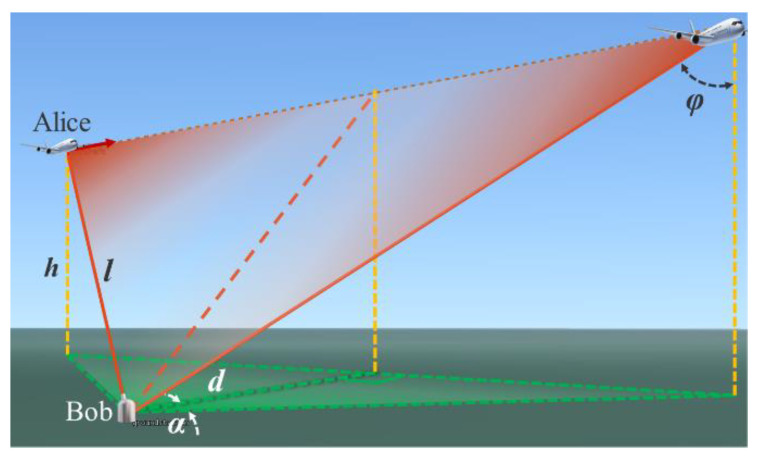
Schematic diagram of airborne QKD downlink.

In the air-to-ground communication scenario of airborne QKD, we assume d is the shortest projection distance to the horizon plane, between the aircraft (Alice) and the receiving ground station (Bob). Alice flies at a constant speed in a certain path obliquely above Bob, as shown in Figure 1. h is the relative height of Alice and Bob which is constant. l is the distance between Alice and Bob. φ is zenith angle. During the entire flight, the azimuth angle α of the aircraft is varying with a range of [−80°, 80°].

### 2.2. Photon Scattering under the Boundary Layer

Given the density field distribution of the boundary layer, the refractive index distribution of the flow field can be calculated by

(1) $$n=1+K_{GD}\cdot \rho ,$$

where *ρ* is the density, and *n* is the refractive index of BL. *K_GD_* is the Gladstone-Dale constent with unit of m^3^/kg. *K_GD_* is usually calculated by the empirical formula

(2) $$K_{GD}=2.23\times 10^{-4}\left(1+\frac{7.52\times 10^{-3}}{\lambda^{2}}\right),$$

where *λ* is the wavelengthof photons with unit of micron. With the settings in Table 1, the G-D coefficient is 2.237 × 10^−4^ m^3^/kg.

**Figure 2:**
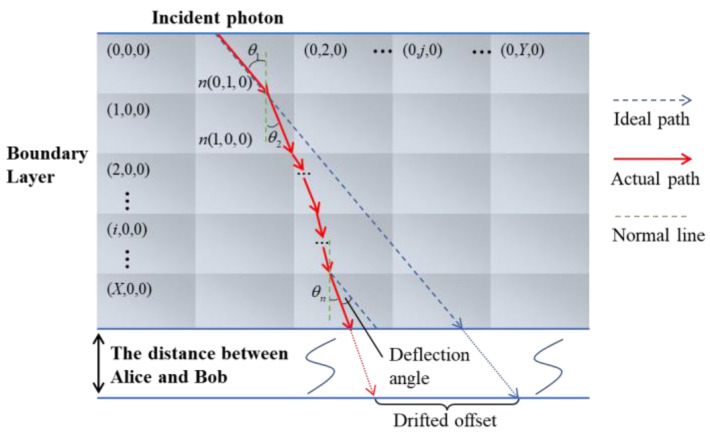
Two-dimensional schematic diagram of the ray tracing method.

The scattered photon path through the boundary layer can be calculated by ray tracing methods [30]. With the varying refractive index, the boundary layer can be divided into several sufficiently small cube units. And the internal refractive index of each unit (i, j, k) is uniform which is n(i, j, k). Then, the photon will be refracted at the boundary of the two grids, as shown in Figure 2, and the new direction of communication can be calculated by Snell law

(3) $$\theta_{2}=\arcsin\left[\frac{n\left(x_{1},\;y_{1},\;z_{1}\right)\sin\left(\theta_{1}\right)}{n\left(x_{2},\;y_{2},\;z_{2}\right)}\right],$$

where $n\left(x_{1},\;y_{1},\;z_{1}\right)$ is the refractive index of unit $\left(x_{1},\;y_{1},\;z_{1}\right)$, $\theta_{1}$ is the incident angle, $\theta_{2}$ is the refraction angle. In the air-to-ground communication scenario, the incident angle $\theta_{1}$ is equal to zenith angle φ which can be calculated as

(4) $$\varphi =\arctan\left(\frac{d}{h\cos\alpha }\right).$$

When the azimuths angle is 0°, the incident angle $\theta_{1}=\varphi =\arctan\left(d/h\right)$. It is worth noting that when the azimuth angle is 0°, the incident angle is not 0°.

And the new incident angle can be calculated by $\theta_{2}$ when the photon propagated to the next grid. Then, recording the whole location $\left(x_{1},\;y_{1},\;z_{1}\right),\;\left(x_{2},\;y_{2},\;z_{2}\right)\ldots \left(x_{n},\;y_{n},\;z_{n}\right)$ by reciprocating the Snell law, the scattered photon path X via the boundary layer can be calculated.

Then, the wavefront information could be calculated. Optical path length (OPL) is calculated by integrating the refractive index *n* along the propagation path X [30].

(5) $$\mathrm{OPL}=\int_{X}ndx.$$

And the optical path difference (OPD) is defined as follows,

(6) OPD=OPL−〈OPL〉.

Afterward, assumed that the photon source conforms to the distribution of the Gaussian beam, the normalized intensity of the Gaussian beam can be expressed as

(7) $$I\left(r,l\right)=\frac{2}{\pi \cdot \omega_{LP}}\exp\left(\frac{-2r^{2}}{\omega_{LP}^{2}}\right),$$

where *r* is the radial distance from the center axis of the beam, *l* is the axial distance from the beam’s focus, and $\omega_{LP}$ is the effective beam waist of the downlink signal at the ground station [31]

(8) $$\omega_{LP}=\sqrt{\omega_{L}{}^{2}+{\left(\sigma_{T}\cdot l\right)}^{2}}.$$

Here, we assumed that the Transmitter pointing precision $\sigma_{T}$ = 150 μrad that is given in [21]. $\omega_{L}$ is the beam waist at the Ground station prior to pointing errors:

(9) $$\omega_{L}=l\frac{\lambda }{\pi \cdot \omega_{0}}{\left[1+0.83\cdot \sec\left(\varphi \right){\left(\frac{D_{T}}{r_{0}}\right)}^{5/3}\right]}^{3/5}.$$

where *r*_0_ = 0.4 m is the Fried parameter in zenith, and φ is zenith angle as shown in Figure 1. *ω*_0_ is the waist radius of the Gaussian beam

(10) $$\omega_{0}=0.316D_{T}.$$

The angled brackets denote the spatial average over the optical aperture.

### 2.3. Transmission Efficiency Analysis

Assuming that the ATP (Acquisition, Tracking, and Pointing) technique is perfect, the center axis of the beam can be aligned with the center of the receiver telescope. And then, the transmission efficiency from aircraft to the ground station can be calculated when the light is via the boundary layer or not.

Without the boundary layer effects, the detected events fulfil the Gaussian distribution. When the beam illuminates the receiving telescope, the transmission efficiency *η*_0_ can be calculated by geometric optics as [31]

(11) $$\eta_{0}=\left[1-\exp\left[-0.5{\left(\frac{D_{R}}{\omega_{LP}}\right)}^{2}\right]\right]\cdot \exp\left[-\beta \cdot \sec\left(\varphi \right)\right],$$

where *D_R_* is the diameter of the receiving telescope, and *β* is the extinction optical thickness between sea level and altitude.

After passing through the boundary layer, the intensity of the beam will be redistributed. Then, by weighting each beam with the Gaussian distribution in Equation (7), the photons distribution probability eventually reaches the receiver telescope will be calculated, when the light is via the boundary layer or not. Then, the photons distribution probability ratio $E_{boundary}$ can be calculated as

(12) $$E_{boundary}=\frac{\varepsilon_{boundary}}{\varepsilon_{0}},$$

where $\varepsilon_{boundary}$ is the statistics of photons distribution probability within the range of receiving telescope per unit time with the effect of BL. While $\varepsilon_{0}$ is the value without the effect of BL.

After passing through the boundary layer, the transmission efficiency $\eta_{boundary}$ can be calculated as

(13) $$\eta_{boundary}=\eta_{0}\cdot \mathrm{SR}\cdot E_{boundary},$$

where SR is the Strehl ratio [32]

(14) $$\mathrm{SR}\approx \exp\left[-{\left(\frac{2\pi {\mathrm{OPD}}_{\mathrm{rms}}}{\lambda }\right)}^{2}\right].$$

where ${\mathrm{OPD}}_{\mathrm{rms}}$ is the RMS of the OPD on the optical aperture.

### 2.4. Secure Key Rate Estimation

The decoy state method [33], as an important weapon to combat photon number splitting attack, is proposed to use a weakly coherent light source in the QKD protocol to replace the ideal single-photon source that cannot be achieved at present. Therefore, the Vacuum + weak BB84 protocol is selected in the airborne QKD system, and a formula for secure key rate is

(15) $$R\geq q\left\{Q_{1}\left[1-H_{2}\left(e_{1}\right)\right]-Q_{\mu }f\left(E_{\mu }\right)H_{2}\left(E_{\mu }\right)\right\}$$

where *Q_1_* is the gain of single-photon states, e_1_ is the error rate of single-photon states, *f*(*x*) is the bidirectional error correction efficiency as a function of error rate, *H_2_*(*x*) is the binary Shannon information function and *μ* is the intensity of the signal state. *Qμ* and *Eμ* respectively represent the gain of signal states and the overall quantum bit error rate.

In free-space quantum communication, it is necessary to consider the reduction of efficiency caused by the diffusion of the light spot at the receiving terminal. With the influence of the boundary layer, the total transmission efficiency is

(16) $$\eta =\eta_{boundary}\eta_{s}\eta_{d}.$$

where *η_s_* is receiving optical module efficiency, and *η_d_* is detector efficiency. Substituting Equations (16) into the formula about Vacuum + weak BB84 protocol in literature [33], and the secure key rate *R* can be calculated.

## 3. Evaluation Result and Discussion

**Figure 3:**
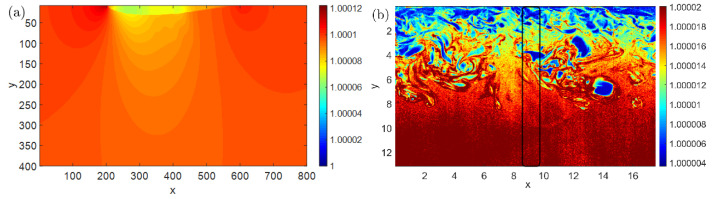
The sectional drawing of refractive index field of (**a**) the airfoil boundary layer and (**b**) the supersonic boundary layer. The coordinate axis represents the size of the boundary layer, in millimeters. The incident points are respectively (x = 340 mm, y = 0) and (x = 9 mm, y = 0). And the color represents the refractive index distribution.

Here, the typical airfoil that named “NACA0015” is chosen for the performance analysis of our specified air-to-ground QKD system. The specific parameter settings of the aircraft, source, ground station and protocols are shown in Table 1. The boundary layer will be generated around the airfoil and its density field distribution can be simulated by the computational fluid dynamics software (Ansys Fluent, Canonsburg, PA, USA) as shown in Figure 3a, with the parameters shown in Table 1. Usually, the boundary layer thickness based on the velocity boundary layer concept that the region in which flow adjusts from zero velocity at the wall to a maximum in the main stream of the flow is termed the boundary layer. According to the simulation results, the airfoil velocity boundary layer thickness is about 10 mm. Here, in order to ensure that the path lengths in the boundary layer are approximately equal and the results are more accurate under different incident angles, we expend the concept of the boundary layer as the field that caused by the motion of aircraft. So, the generalized boundary layer thickness is 400 mm.

Literature [34] used the nano-particle-based planar laser scattering technique to measure the density distribution of the supersonic (Ma = 3.0) turbulent boundary layers, as shown in Figure 3b. The velocity boundary layer thickness is about 10 mm [33], and the generalized boundary layer thickness is about 13 mm. Although this kind of boundary layer is not the actual aircraft boundary layer, the turbulence structure is similar to the actual situation. Due to the limited amount of data, we only analyze the results at a certain moment, without considering an average over enough runs. It is also meaningful to research the influence of this kind of boundary layer on QKD performance.

**Figure 4:**
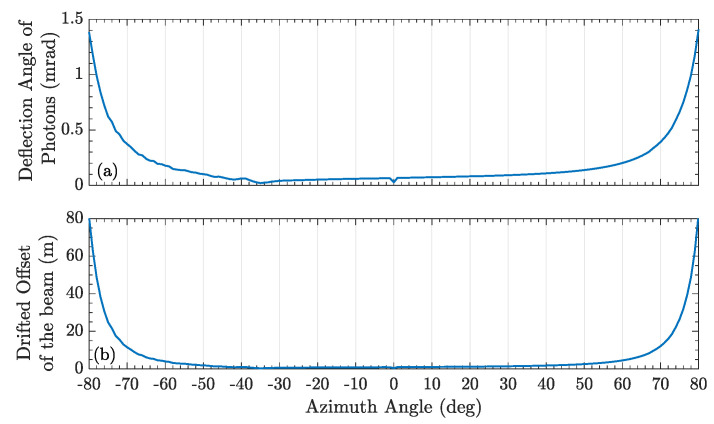
(**a**) The deflection angle of transmitted photons. (**b**) The drifted offset of the beam, which reaches to the ground station. The speed of airborne is 0.7 Ma.

**Figure 5:**
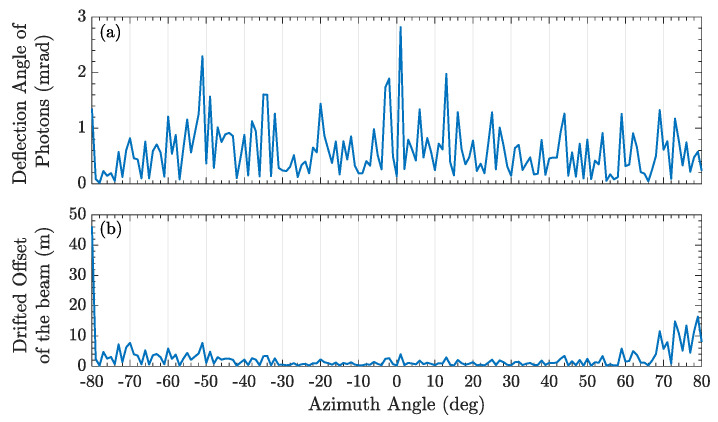
(**a**) The deflection angle of transmitted photons. (**b**) The drifted offset of the beam, which via the supersonic boundary layer and reaches the ground station.

When the photon trajectories of different incident angles pass through the boundary layer and reached the ground station, the evaluated deflection angle and the drifted offset of the beam are shown in Figure 2. The deflection angle can be calculated as $\left|\theta_{1}-\theta_{n}\right|$ which is absolute value of the angle difference between the incident angle $\theta_{1}$ and the last refracted angle $\theta_{n}$ when the photon propagated via the boundary layer. In both cases, the deflection angle and correspondingly drifted offset of the beam were varied obviously in Figure 4 and Figure 5. Because of the complex small-scale turbulence structures in the supersonic boundary layer, the spatial distribution of the density field is anisotropic and random, and the results vary randomly as shown in Figure 5. When the azimuth angle is near zero, the incident photon will pass through the region with a large refractive index gradient, as shown in Figure 3b, which could influence the deflection angle obviously. In a practical airborne quantum communication system, owing to the ATP technique, the center axis of the beam can always be aligned with the center of the receiver telescope. Therefore, the deflection angle and the drifted offset were not analyzed emphatically in this paper, which could been ignored.

**Figure 6:**
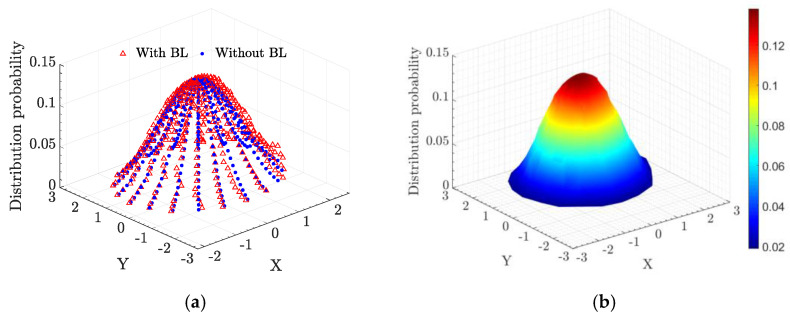
(**a**) The photons distribution probability when the photons via the supersonic BL or not, which are denoted by a red triangle and blue point respectively. (**b**) The photons distribution probability when the photons via the supersonic BL. The azimuth angle is 10°.

Afterward, the photons distribution probability that eventually reaches the receiver telescope at azimuth angle is 10°,was calculated as shown in Figure 6, when the photon via the supersonic BL or not. It shows that the photons distribution probability is redistributed when the scattered photon passes through the supersonic BL. But the photons distribution probability is fundamentally invariant when the photon via the BL when flight speed is 0.7 Ma, which is not displayed.

**Figure 7:**
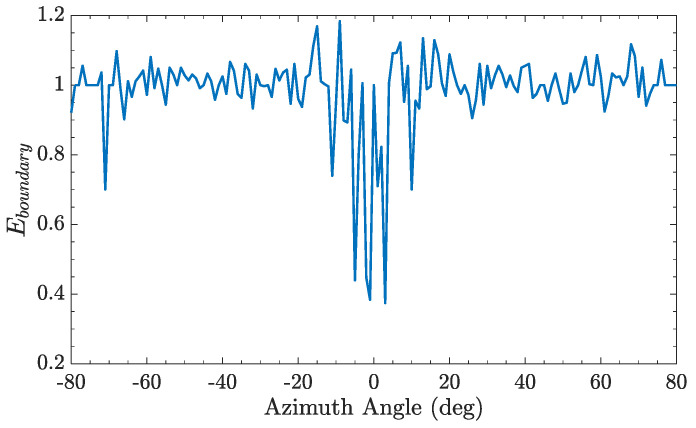
The receiving photons distribution probability ratio over the azimuth angle, when the photon via the supersonic BL.

Then, the receiving photons distribution probability ratio $E_{boundary}$ in Equation (12) over the azimuth angle can be calculated, as shown in Figure 7, when the photon via the supersonic BL. The curve random jitter is more obvious, and $E_{boundary}>1$ at some azimuth angle which shows that photons bunching has happened. Moreover, when the refractive index gradient is less than zero, the refraction angle is larger than incident angle. In this case, the photon spot dispersion caused by the small incident angle is more serious due to the limited number of grids. As shown in Figure 4, The small incidence angle will cause the beam to separate to the two sides, intensify the diffusion of light spots, and reduce the receiving photons distribution probability ratio $E_{boundary}$. The case of the small incident angle with the negative refractive index gradient is indicated by the black box in Figure 3b. So, it is possible that significant dispersion phenomenon occurs around the zero azimuthal angle.

**Figure 8:**
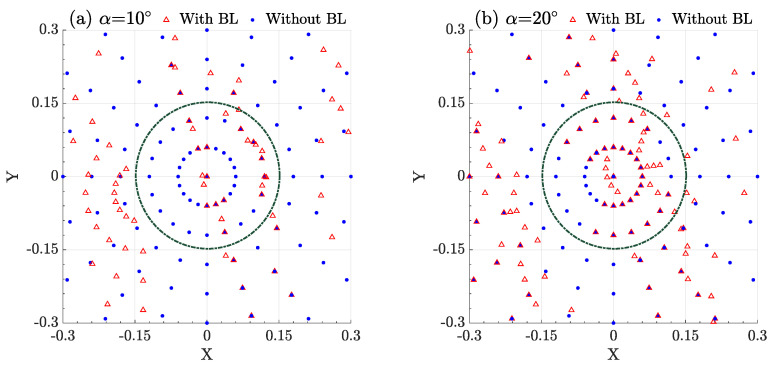
(**a**) The receiving photons distribution when the azimuth angle is 10° with and without the effect of supersonic BL, which are denoted by a red triangle and blue point respectively. (**b**) The receiving photons distribution when the azimuth angle is 20° with and without the effect of supersonic BL. The green dotted line indicates the aperture of the receiving telescope.

When Figure 6 is converted into a top view, as shown in Figure 8, the distribution of photons can be well displayed with and without the effect of supersonic BL. By comparing the two cases where the azimuth is 10° and 20°, the probability of photon appearing in the range of the receiving telescope after passing through the boundary layer is different. When the azimuth is 10°, the photons passe through the boundary layer and are partially refracted out of the scope of the telescope. When the azimuth is 20°, some photons that are not in the scope of the telescope are refracted into the telescope after passing through the boundary layer. By contrast, the case of $E_{boundary}>1$ can be explained.

**Figure 9:**
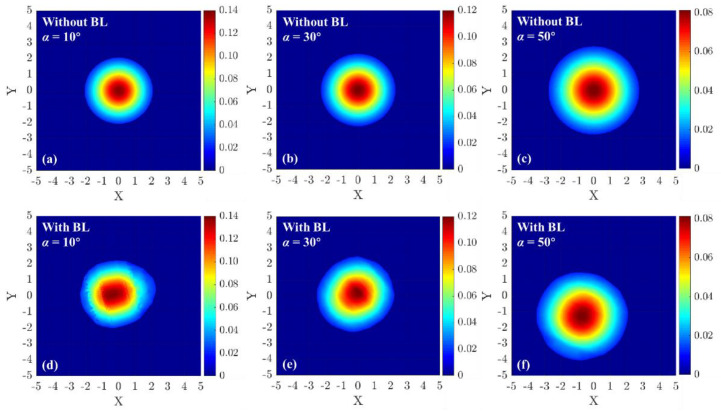
The photons distribution probability with different azimuth when the photon via the boundary layer or not. (**a**–**c**) show the photons distribution probability of the Gaussian beam without BL. (**d**–**f**) show the photons distribution probability of the Gaussian beam that passes through the BL. (**a**,**d**) show the situation when the azimuth angle is 10°. (**b**,**e**) show the situation that the azimuth angle is 30°. (**c**,**f**) show the situation the azimuth angle is 50°.

And the photons distribution probability with several different azimuth angles can be calculated, when the photons via the supersonic BL or not. As shown in Figure 9, different from the subsonic boundary layer, the supersonic boundary layer can diffuse the probability of photons distribution. It can cause the photon of receiving terminal to be so irregular that the transmission efficiency and secure key rate decrease.

**Figure 10:**
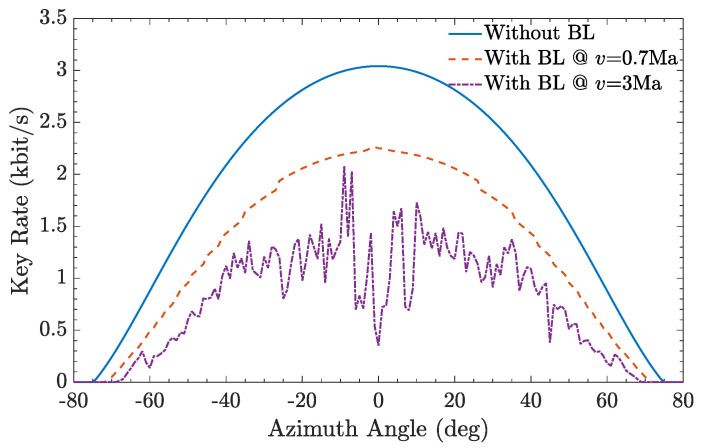
The secure key rate over the azimuth angle when the photon via subsonic BL, supersonic BL, or not.

By taking the total transmission efficiency into the formula of the key rate, the QKD performance with the BL effects is evaluated and the result is shown in Figure 10. When the photon via the BL, the key rate curve drops obviously. Compared with the subsonic surface layer, the effect of the supersonic surface layer is more obvious. The curve varies even more dramatically, and the effect of supersonic BL at individual points is very obvious. Therefore, the estimated average secure key rate is around 943 bit/s and 551 bit/s respectively when the photon via the subsonic and supersonic surface layer. If there’s no boundary layer surrounding the aircraft, the estimated average secure key rate would be around 1468 bit/s. It shows that the actual supersonic BL can also have an impact on the QKD performance, and the effect is more obvious. In addition, due to the higher speed, the effective communication time between the vehicle and the ground station will be shorter, and the total key rates will be smaller. Further, although the impact of the deflection angle and the drifted offset could be ignored, some potential factors will also affect the performance of QKD which from ATP system. Therefore, it is feasible to implement QKD by supersonic aircraft, but the results would be unsatisfactory.

## 4. Conclusions

Airborne quantum key distribution (QKD) that can synergize with terrestrial networks and quantum satellite nodes is expected to provide flexible and relay links for the large-scale integrated communication network. However, the photon transmission rate would be randomly reduced, owing to the random distributed boundary layer that surrounding to the surface of the aircraft when the flight speed larger than Mach 0.3, which would change the local refractive index and energy flux density drastically. Different from satellite based implementations, airborne platforms need to consider the influence of boundary layer due to the atmosphere. In this article, an airborne QKD performance evaluation scheme with supersonic BL effect is proposed. Through modeling and analysis, owing to the complex small-scale turbulence structures in the supersonic boundary layer, the deflection angle and correspondingly drifted offset of the beam varied obviously and randomly, and the distribution probability of photons are redistributed. The result shows that the subsonic and supersonic boundary layer would decrease ~35.8% and ~62.5% of the secure key rate respectively. Our work provides a theoretical guidance towards a possible realization of high-speed airborne QKD.

## Figures and Tables

**Table 1:** List of parameters and values for which we assigned fixed values.

	Symbol	Parameter	Value
Aircraft	*v*	Flight speed	0.7 Ma
	*h*	Relative flying height (*h = h_T_* − *h_R_*)	10 km
	*ρh*	Air density	0.41271 kg/m^3^
	*d*	The shortest projection distance to the ground of the aircraft and the ground station	10 km
Source	*h_T_*	Altitude of the aircraft	11 km
	*D_T_*	Transmitter telescope diameter	0.1 m
	*δ_T_*	Transmitter pointing precision	2.4 μrad
	*λ*	Transmitter wavelength	1550 mm
	*ω_0_*	Waist radius	0.0316 m
Ground station	*h_R_*	Altitude of the ground station	1 km
	*D_R_*	Receiver telescope diameter	0.3 m
	*e_d_*	Detection error rate	1%
	*p_d_*	Dark count	2 × 10^−6^
	*η_d_*	Detector efficiency	15%
	*η_s_*	Receiving optical module efficiency	60%
Protocols	*μ*	Expected photon number of signal states	0.1
	*ν*	Expected photon number of decoy states	0.05
	*N*	System repetition rate	100 MHz
	*Ps*	Sent Probability of signal states	50%
	*Pd*	Sent Probability of decoy states	25%

## Data Availability

Not applicable.
